# Supplementary material for: Selecting optimal software code descriptors—The case of Java
Source: PLoS One. 2024 Nov 1;19(11):e0310840. doi: 10.1371/journal.pone.0310840 (PMC11530023; doi:10.1371/journal.pone.0310840)
Supplement: S1 Appendix — (PDF) [file pone.0310840.s001.pdf]

A Minimization of Sammon error

Table A1. Results of PSO with Sammon error for metrics at the class level

	2	3	4	5	6	7	8	9	10	11	12	13	14	15	16	17	18	19	20
	0.55	0.43	0.39	0.31	0.28	0.22	0.19	0.18	0.15	0.13	0.11	0.09	0.08	0.06	0.06	0.05	0.04	0.03	0.03
CD	266	341	407	446	486	521	550	569	589	631	623	654	676	693	709	719	733	741	747
NOP	195	258	308	332	374	405	418	462	482	477	514	539	551	560	582	582	608	620	619
NLE	122	187	241	300	341	385	418	469	471	503	542	559	606	610	652	668	683	704	726
DIT	118	166	219	242	265	327	336	384	413	440	465	484	506	543	553	576	589	595	631
CBO	88	146	190	238	285	318	367	397	451	469	482	528	559	578	583	628	638	664	685
NL	80	156	192	227	265	301	325	362	406	438	465	490	524	555	572	601	626	645	683
NA	74	98	140	197	224	283	312	353	414	426	453	490	511	533	569	589	598	623	656
TNA	63	108	158	204	238	249	325	337	353	403	433	457	488	523	559	569	589	620	634
NOA	82	109	136	177	227	240	280	308	327	364	399	421	457	472	497	511	540	556	605
CBOI	42	67	98	128	156	198	235	275	295	322	383	407	464	472	507	535	556	610	630
NM	29	60	85	109	135	163	224	239	270	316	340	352	402	427	462	488	535	551	562
NPM	33	70	92	113	160	178	208	241	272	282	337	378	388	415	463	478	504	547	558
LCOM5	29	55	89	113	147	175	210	242	273	310	335	359	402	428	439	480	516	520	567
RFC	46	66	100	131	149	184	182	230	260	292	319	359	396	413	466	473	516	548	558
TNPM	44	62	79	113	149	174	211	227	252	290	312	342	350	421	420	445	494	500	551
TNM	43	59	78	115	137	176	195	219	251	305	314	327	360	379	442	463	483	512	549
NOI	27	51	71	95	130	153	176	218	247	262	302	315	346	399	400	454	480	513	539
TLLOC	25	39	55	77	94	129	141	171	192	256	255	302	324	361	384	412	455	512	497
TLOC	20	40	47	84	86	120	156	157	207	226	266	289	303	332	381	418	420	473	514
LOC	13	27	44	57	94	103	127	161	175	173	221	264	287	324	342	393	436	437	518
TNOS	26	35	59	81	93	120	146	170	216	221	253	283	293	337	395	396	412	459	486
WMC	35	36	72	66	89	109	152	174	184	224	249	273	308	337	337	394	428	446	489
CLOC	27	38	37	61	86	100	108	127	156	171	199	226	234	276	304	315	351	383	420
TNPA	19	18	24	40	52	79	91	106	132	139	175	187	218	225	244	281	303	329	351
NOS	18	38	49	68	76	86	131	150	170	189	213	258	269	314	319	364	414	429	447
NII	17	24	47	74	87	110	106	129	150	185	203	240	264	309	334	348	406	428	475
LLOC	12	28	49	65	92	99	126	156	178	210	239	258	311	307	354	410	412	464	477
NPA	5	13	23	25	40	62	72	76	89	120	129	152	154	183	199	226	250	285	299
NOD	2	2	5	8	20	27	34	46	70	77	95	123	135	150	184	202	217	241	262
NOC	0	3	6	14	23	26	38	45	55	79	85	84	114	124	148	182	208	245	265

Table A2. Results of GA with Sammon error for metrics at the class level

Metric	2	3	4	5	6	7	8	9	10	11	12	13	14	15	16	17	18	19	20
	0.55	0.43	0.39	0.31	0.28	0.22	0.19	0.18	0.15	0.13	0.11	0.09	0.08	0.06	0.05	0.05	0.04	0.03	0.03
CD	303	377	403	439	486	519	539	586	599	609	629	650	677	697	704	691	732	740	749
NOP	236	257	303	349	367	418	417	436	453	483	496	527	541	537	559	602	596	631	627
NLE	129	189	277	314	355	409	428	456	482	506	531	565	596	616	628	670	676	680	704
DIT	116	163	208	238	287	303	355	354	404	427	463	472	497	505	539	570	589	613	623
CBO	101	160	193	246	289	322	359	380	414	434	467	496	509	553	582	615	627	649	662
NL	89	162	174	234	241	289	338	352	401	410	459	487	489	528	554	578	623	637	663
NA	59	106	151	176	224	245	282	296	350	375	413	440	469	502	525	543	566	588	629
TNA	62	92	138	184	197	232	271	307	306	351	399	434	450	485	492	518	562	589	618
NOA	71	127	150	167	206	220	253	303	316	360	382	417	442	471	490	500	533	549	590
CBOI	43	62	105	143	175	212	245	289	302	329	355	397	425	454	498	526	559	606	610
NOI	25	53	78	119	143	172	206	223	249	310	310	352	369	411	440	470	495	519	555
LCOM5	39	65	91	125	173	179	211	240	261	308	368	372	426	422	474	518	543	554	592
NPM	29	47	87	122	133	183	222	245	271	297	347	359	384	406	451	468	499	539	543
RFC	61	80	104	104	149	176	198	237	283	291	310	309	400	421	445	454	499	513	568
TNPM	27	53	82	110	142	163	187	220	217	254	280	324	339	417	405	454	466	500	542
NM	26	50	77	122	149	161	192	240	255	274	320	342	351	417	454	459	504	530	566
TNM	20	47	60	86	98	157	175	204	251	272	305	332	364	369	416	446	497	487	517
TLLOC	16	24	59	73	98	126	142	182	232	233	259	293	325	368	376	376	447	477	486
LOC	12	24	45	68	87	116	140	188	193	236	260	291	339	349	344	394	430	467	501
TLOC	10	30	54	81	91	123	148	175	216	220	238	289	303	324	375	420	409	448	498
LLOC	9	21	48	67	98	127	132	179	186	241	266	286	326	334	379	398	415	467	493
WMC	32	50	65	73	95	134	173	170	195	248	263	264	292	333	360	411	420	450	470
CLOC	27	31	50	68	91	114	121	155	163	199	230	246	277	311	332	329	394	411	443
TNOS	20	36	47	77	98	99	144	171	194	209	250	276	317	322	357	384	436	462	464
NII	17	32	47	67	89	109	134	158	201	231	235	274	285	318	344	394	407	460	473
NOS	12	36	49	59	95	118	141	147	199	227	241	264	297	330	348	390	385	440	483
TNPA	4	15	20	33	51	47	78	85	132	138	159	194	206	243	271	289	295	344	382
NPA	4	8	20	32	50	62	70	101	109	140	148	172	208	208	244	275	300	302	334
NOD	1	1	6	17	21	38	48	56	91	87	103	132	157	165	209	217	257	282	311
NOC	0	2	9	7	22	27	51	65	75	101	114	144	140	184	205	241	239	266	304

Table A3. Results of PSO with Sammon error for metrics at the method level

Metric	2	3	4	5	6	7	8	9	10	11	12	13	14	15	16	17	18	19	20
	0.50	0.44	0.33	0.26	0.23	0.18	0.15	0.12	0.10	0.09	0.07	0.06	0.05	0.04	0.03	0.03	0.02	0.02	0.01
NUMPAR	319	433	519	564	615	652	673	681	709	732	738	746	763	768	775	786	786	791	794
CD	259	302	362	442	469	507	550	573	583	623	645	645	672	673	693	700	713	726	739
TCD	115	227	324	367	450	489	522	545	569	602	596	642	650	668	670	703	704	728	737
NLE	86	176	251	328	389	439	453	530	551	598	609	633	653	665	697	708	727	732	748
NOI	76	134	189	255	305	348	380	422	468	490	513	539	556	588	613	626	647	669	686
NL	73	121	174	210	273	322	365	394	439	472	505	533	570	608	618	636	659	683	704
HDIF	103	142	179	232	262	331	372	419	447	483	504	559	565	615	626	656	673	702	714
MI	115	138	167	217	234	286	320	349	395	422	469	487	526	585	612	621	678	704	737
HPV	67	101	138	187	217	270	313	390	413	437	479	531	554	595	623	636	670	676	714
NII	49	81	121	167	210	262	297	327	366	409	446	484	525	567	596	633	667	680	694
HCPL	26	45	73	112	150	173	223	241	289	332	375	418	449	486	538	558	578	620	642
TNOS	31	52	71	100	106	141	190	225	264	301	331	372	413	447	477	532	550	575	626
HPL	29	34	48	67	103	121	160	181	237	277	316	381	413	447	460	508	545	583	620
NOS	40	43	70	91	109	146	174	201	244	285	329	366	401	433	470	534	531	584	602
TLLOC	23	49	70	81	118	141	179	201	247	269	320	332	397	406	441	486	526	563	589
TLOC	49	54	74	84	105	129	147	205	234	271	303	344	370	403	461	511	529	590	600
LOC	27	42	61	65	80	116	150	179	217	243	274	316	354	384	437	464	526	542	581
HNDB	10	22	29	39	49	66	98	114	142	162	203	233	263	291	343	409	460	460	521
LLOC	20	23	36	53	75	74	118	150	173	194	242	286	334	369	405	431	443	492	544
TCLOC	20	48	54	70	91	117	127	159	194	215	238	270	308	335	366	407	434	469	518
DLOC	40	77	102	110	153	166	207	216	236	256	279	305	337	360	375	398	425	467	498
CLOC	10	22	31	62	80	90	121	152	173	204	238	252	293	308	349	371	435	458	480
HVOL	7	19	29	36	48	61	86	105	129	163	196	217	255	317	346	379	415	462	518
McCC	3	7	17	25	58	62	77	100	108	137	164	193	213	246	272	310	374	382	420
HTRP	1	4	4	8	12	21	20	29	33	53	60	71	84	100	120	144	170	237	257
LDC	1	3	1	10	12	23	24	38	48	66	80	88	100	117	136	160	180	199	232
HEFF	1	0	2	10	14	23	19	31	43	46	82	79	86	99	137	141	181	220	260
LLDC	0	1	4	8	13	24	35	43	49	58	66	78	96	120	144	152	174	206	225

Table A4. Results of GA with Sammon error for metrics at the method level

Metric	2	3	4	5	6	7	8	9	10	11	12	13	14	15	16	17	18	19	20
	0.50	0.44	0.33	0.26	0.23	0.18	0.15	0.13	0.10	0.09	0.07	0.06	0.05	0.04	0.03	0.03	0.02	0.02	0.01
CD	315	315	323	383	428	463	485	524	558	560	572	619	638	658	687	683	710	716	729
NUMPAR	308	423	472	537	566	623	646	656	682	706	725	734	742	764	763	780	786	791	795
TCD	90	235	332	365	407	456	476	507	531	583	592	621	628	654	658	694	714	721	739
NLE	111	191	279	315	339	404	407	463	497	532	565	580	603	622	672	683	701	719	734
NOI	88	149	194	234	303	324	350	385	429	455	506	521	544	574	599	614	631	646	681
NL	89	144	180	222	251	283	321	370	390	425	464	494	531	570	593	599	634	654	701
HPV	68	91	135	175	214	274	277	297	341	393	425	453	499	514	565	591	604	634	664
HDIF	125	158	200	216	244	257	326	354	377	426	456	491	525	563	580	613	638	658	701
MI	126	150	188	203	220	253	303	344	375	413	438	490	509	550	583	609	661	698	733
NII	51	89	122	168	208	235	294	324	360	400	430	461	519	548	587	623	661	684	707
HCPL	32	63	92	117	159	197	220	274	311	313	360	378	449	470	479	499	566	601	648
TNOS	27	43	72	100	137	160	177	216	269	291	324	349	364	405	462	510	528	562	593
NOS	15	27	59	91	136	133	185	225	223	285	285	341	395	411	453	494	505	552	590
TLOC	8	30	53	83	109	131	153	176	220	252	318	330	381	412	430	484	504	544	566
HPL	25	35	56	78	110	129	172	215	244	274	311	321	371	403	417	462	511	551	567
TLLOC	9	23	59	84	119	161	185	213	239	271	302	341	361	401	466	467	512	544	566
LOC	13	30	42	87	112	140	162	198	228	255	290	317	349	387	414	466	468	544	555
HVOL	4	17	30	64	82	128	162	170	204	219	266	296	312	343	362	411	466	483	512
DLOC	40	74	98	121	150	169	204	218	229	258	284	329	361	380	405	427	459	499	495
LLOC	12	21	37	61	105	128	157	172	223	243	261	306	341	364	392	439	449	489	532
TCLOC	18	27	37	79	90	111	149	165	203	249	250	274	306	362	370	372	440	465	531
HNDB	10	16	37	56	74	106	142	173	191	224	249	282	283	335	377	417	424	451	515
CLOC	9	30	50	61	86	108	140	161	194	217	240	283	293	323	380	412	425	473	507
McCC	4	11	23	42	55	85	105	118	149	165	201	234	266	295	326	355	403	446	464
HEFF	2	1	7	22	37	48	69	91	99	106	132	147	190	184	228	249	285	311	343
LDC	1	1	8	11	18	31	38	57	79	84	108	110	138	140	175	207	224	235	251
LLDC	0	3	8	9	16	29	42	52	69	84	108	114	128	153	166	177	213	220	259
HTRP	0	3	7	16	25	34	53	82	86	117	138	184	174	215	211	263	278	309	322
