# Supplementary material for: Selecting optimal software code descriptors—The case of Java
Source: PLoS One. 2024 Nov 1;19(11):e0310840. doi: 10.1371/journal.pone.0310840 (PMC11530023; doi:10.1371/journal.pone.0310840)
Supplement: Attachment — Submitted filename: response_letter.pdf [file pone.0310840.s002.pdf]

Responses to the reviewers of the paper

Selecting Optimal Software Code Descriptors – the Case of Java

Submitted to Plos One

Dear Sirs,

We would like to express our most sincere gratitude to the reviewers for the very valuable observations that they provided us, which allowed us to identify weak areas in our work and, hopefully, to improve it.

In the remaining of this document there are our specific responses to them.

Best regards,

Yegor Bugayenko, Zamira Kholmatova, Artem Kruglov, Witold Pedrycz, and Giancarlo Succi

Reviewer 1

Comment 1.1 They applied particle swarm optimization (PSO) and a genetic algorithm (GA). However, good results have also been obtained with the techniques described in https://doi.org/10.1016/j.asoc.2023.110659. If the authors offer a solution that does not minimize the prediction error, it would be interesting for readers to see a comparison of the proposed solution with those mentioned in the paper.

Response to Comment 1.1 Thank you for bringing this paper to our attention. We have compared our results with those presented in the suggested work and included this comparison in the Conclusion. They have confirmed that nesting level, depth of inheritance tree, and the number of dependencies that the observed class owns – the metrics that also appeared in our optimal subsets, are significant for the defect prediction.

Comment 1.2 How were the 1000 repositories to be analyzed determined? How was this value chosen, and does it affect the results obtained?

Response to Comment 1.2 Thank you for your insightful question. The collected repositories constitute 4.5% of the total number of open and unarchived repositories with at least 100 stars. Moreover, these repositories are related to popular topics on GitHub like “java”, “spring-boot”, “redis”, “docker”, and “machine-learning”. This information we have added to the Data collection (Ssection 3.1).

Comment 1.3 Sections 3.3.1 and 3.3.2 contain details about algorithms that are likely familiar to readers, so it is recommended to shorten them.

Response to Comment 1.3 Thank you for this helpful remark. We have shortened Sections 3.3.1 and 3.3.2 as suggested.

Comment 1.4 Are the results affected by the choice of programming language? How do the characteristics of the programming language impact the research? In the conclusion, the authors mention the application of their work with other programming languages, but a more detailed analysis would be useful.

Response to Comment 1.4 Thank you for your comment. We have highlighted the directions for future work in the Conclusion.

We assume that other languages similar to Java (for example, C#) will work similarly, but at the moment we have no supporting evidence. Therefore to understand the effect of the choice of programming languages on the results we want to proceed with experiments on other languages.

Reviewer 2

Comment 2.1 Describe dataset features in more details and its total size and size of (train/test) as a table.

Response to Comment 2.1 Thank you very much for the remark. We have added Table 3 with the details on the train/test sets.

Comment 2.2 Pseudocode / Flowchart and algorithm steps of the proposed method need to be inserted.

Response to Comment 2.2 Thank you for the comment that helps to improve the readability of our work. We have added Figure 1, explaining the proposed methodology.

Comment 2.3 Time spent need to be measured in the experimental results.

Response to Comment 2.3 Thank you for the suggestion. We have added Table 5 with the statistics on the time spent for our experiments.

Comment 2.4 Limitation and Discussion Sections need to be inserted.

Response to Comment 2.4 Thank you for the valuable comment. Now, the readers can see the Discussion (Section 5) and Limitation (Section 7).

Comment 2.5 The parameters used for the analysis must be provided in table.

Response to Comment 2.5 We have added Table 4 with the experimentation parameters of PSO and GA to address your suggestion.

Comment 2.6 The architecture of the proposed model must be provided

Response to Comment 2.6 The architecture of the proposed model is now provided in a flowchart in Figure 1.

Comment 2.7 Address the accuracy/improvement percentages in the abstract and in the conclusion sections, as well as the significance of these results

Response to Comment 2.7 Your feedback is much appreciated. We have included information about the accuracy/improvement percentages and the significance of these results in the Abstract and Conclusion. The optimal subsets identified by our method yielded low values of Sammon error in 74% of validation repositories at the class level and 73% at the method level.

Comment 2.8 The authors need to make a clear proofread to avoid grammatical mistakes and typo errors

Response to Comment 2.8 The manuscript has undergone a thorough proofreading and grammatical check using digital technologies.

Comment 2.9 Add future work in last section (conclusion) (if any).

Response to Comment 2.9 Thank you for the comment. We have added our plans on the future work to the last section.

Comment 2.10 The authors need to add recent articles in related work and update them.

To improve the Related Work and Introduction sections authors are recommended to review this highly related research work paper:

1. An Approach to Slicing Object-Oriented Programs;
2. An ASP .NET Web Applications Data Flow Testing Approach;
3. Automatic PSO Based Path Generation Technique for Data Flow Coverage;
4. A new feature selection method based on frequent and associated itemsets for text classification;
5. Topic Extraction and Interactive Knowledge Graphs for Learning Resources;
6. Developing an efficient method for automatic threshold detection based on hybrid feature selection approach.

Response to Comment 2.10 We have reviewed the very valuable suggested studies and incorporated all of them into the suitable paragraphs of the section about the state of the art.
